# Workplace Intervention for Heat Stress: Essential Elements of Design, Implementation, and Assessment

**DOI:** 10.3390/ijerph19073779

**Published:** 2022-03-22

**Authors:** Jason Glaser, David H. Wegman, Esteban Arias-Monge, Felipe Pacheco-Zenteno, Heath Prince, Denis Chavarria, William Jose Martinez-Cuadra, Kristina Jakobsson, Erik Hansson, Rebekah A. I. Lucas, Ilana Weiss, Catharina Wesseling

**Affiliations:** 1 La Isla Network, 2219 California Ave NW, #52, Washington, DC 20008, USA; esteban@laislanetwork.org (E.A.-M.); william@laislanetwork.org (W.J.M.-C.); kristina.jakobsson@amm.gu.se (K.J.); erik.hansson@amm.gu.se (E.H.); r.a.i.lucas@bham.ac.uk (R.A.I.L.); ilana@laislafoundation.org (I.W.); ineke@laislanetwork.org (C.W.); 2 University of Massachusetts Lowell, Lowell, MA 01854, USA; 3 Unidad de Gestión Ambiental y Seguridad Laboral, Instituto Tecnológico de Costa Rica, 15th Street, 14th Avenue, Cartago 159-7050, Costa Rica; 4 School of Public Health and Community Medicine, Sahlgrenska Academy, University of Gothenburg, 405 30 Göteborg, Sweden; guspachelu@student.gu.se; 5 Ray Marshall Center, LBJ School of Public Affairs, University of Texas at Austin, 3001 Lake Austin Blvd., Ste. 3.200, Austin, TX 78703, USA; heath.prince@austin.utexas.edu; 6 Occupational Health, Ingenio San Antonio, Chinandega 26100, Nicaragua; dchavarria@sersanantonio.com; 7 Occupational and Environmental Medicine, Sahlgrenska University Hospital, 405 30 Göteborg, Sweden; 8 School of Sport, Exercise and Rehabilitation Sciences, University of Birmingham, Edgbaston, Birmingham B15 2TT, UK; 9 Unit of Occupational Medicine, Institute of Environmental Medicine, Karolinska Institutet, Solnavägen 4, 113 65 Stockholm, Sweden

**Keywords:** chronic kidney disease, heat stress, workplace intervention, occupational health implementation, acute kidney injury, agriculture, occupational exposure, effectiveness, rest-shade-water, occupational health

## Abstract

Heat stress is associated with numerous health effects that potentially harm workers, especially in a warming world. This investigation occurred in a setting where laborers are confronted with occupational heat stress from physically demanding work in high environmental temperatures. Collaboration with a major Nicaraguan sugarcane producer offered the opportunity to study interventions to prevent occupational heat-stress-related kidney disease. Two aims for this study of a rest-shade-water intervention program were: (1) describe the evolving intervention, summarize findings that motivated proposed improvements, assess impact of those improvements, and identify challenges to successful implementation and (2) extract primary lessons learned about intervention research that have both general relevance to investigations of work-related disease prevention and specific relevance to this setting. The learning curve for the various stakeholders as well as the barriers to success demonstrate that effectiveness of an intervention cannot be adequately assessed without considerations of implementation. Designing, effectively implementing, and assessing both health impacts and implementation quality is a resource-intensive endeavor requiring a transdisciplinary approach. Both general and specific lessons learned are presented for decisions on study design and study elements, implementation assessment, and management engagement in understanding how productivity and health can be successfully balanced and for building effective communication between investigators and all levels of management.

## 1. Introduction

Many laborers in lowland Mesoamerica are confronted with occupational heat stress from the combination of physically demanding work that is carried out in high environmental temperatures [1,2,3,4,5,6,7]. Heat stress has multiple well-established adverse health outcomes, including increased accidents, heart attacks, heat illness, and stroke, all potentially fatal and all associated with decreased productivity. Heat stress and its health effects harm workers, and hence, employers requiring active intervention in a warming world [8,9,10,11,12,13].

A more recently recognized health risk from occupational heat stress is acute and chronic kidney disease [1,2,3,4,5]. Chronic kidney disease of non-traditional origin (CKDnt) is a global public health problem, with epidemics identified in Mesoamerica, Sri Lanka, India, and other tropical regions [2,3,4]. Since 1990, CKDnt has accounted for tens of thousands of deaths among working-age people in Mesoamerica, overwhelming health care systems. Sugarcane workers with demanding physical workloads in very hot climatic conditions are the most affected [1,2,3,4,5]. It has been shown that manual sugarcane cutters with the highest levels of work demands have the highest risk of kidney injury and disproportionately suffer from CKDnt [14,15]. In 2012, a study in Chichigalpa, Nicaragua, a community dependent on sugarcane work, found the prevalence of reduced kidney function (eGFR < 60) among men was as high as 42% [1]. Other industries with high prevalence of reduced kidney function include construction, mining, brick production, and grape harvesting [16,17,18,19,20]. These settings have in common physically demanding work, high environmental temperature, and historically poor labor protections.

The association of CKDnt with heat merits response. Protecting the health and livelihood of these workers through the development, implementation, and evaluation of feasible and adaptable workplace interventions should be a priority. For the most part, current at-risk workers are employed in locations where few, if any, preventative measures have been implemented.

A recent review found that only 22% of publications on interventions assessed health outcomes [21], illustrating the need for increased efforts to properly assess the impact of occupational health-interventions efforts.

This report presents the evolution of a large intervention effort designed to reduce heat stress risk for manual laborers exposed to unusually high heat stress from the combination of external and internally generated heat while engaged in sugarcane agriculture. There are two aims: (1) describe the intervention as it evolved by summarizing the findings that motivated proposed improvements along with the impact assessment of those improvements highlighting the challenges to successful implementation and (2) identify the primary lessons learned about intervention research that have both general relevance to investigations on prevention of work-related disease and specific relevance to similar agricultural settings.

## 2. Overview

The intervention assessment reported here occurred at the Ingenio San Antonio (ISA) sugarcane mill in Chinandega, Nicaragua. This sugar mill had, for more than a decade, implemented a pre-employment screening program designed to hire only individuals with good kidney function. Manual laborers continued to suffer kidney damage, which in turn led to the addition of a kidney CKDnt-prevention program based on principles of heat stress relief promoted by the U.S. Occupational Safety and Health Administration program [22].

In 2017, the German Development Bank provided a loan to Ingenio San Antonio (ISA) for incorporating in their development agenda the prioritization of occupational safety and health in an effort to limit kidney injury and subsequent disease through an intervention improving workplace protections. La Isla Network was engaged to carry out an external evaluation of the ISA’s existing prevention program and to recommend enhancements if needed—the Adelante Initiative.

We present here an overview of the existing assessment of kidney health at the mill, the design of the recommended enhancements to the prevention program, the design of the program assessment methods, and the results to date in terms of kidney health and in economic terms directly relevant to sustainability. We conclude with lessons both general and specific concerning intervention efforts to prevent work-related disease.

## 3. The Intervention

The foundation for the evolving intervention program is described in detail in publications regarding the pilot study of efficacy in El Salvador [23,24] and as adapted for use in the current Nicaraguan setting [14,15]. This program has now been systematized in the transdisciplinary Prevention, Resilience, Efficiency and Protection (PREP) program [25]. The program was designed with the understanding that practical intervention continuously needs to address:

- Successful engagement of stakeholders at all levels,
- A learning curve for all involved,
- Continuous adaptation that characterizes any ongoing operation,
- Formal assessment of effectiveness using agreed-upon metrics, and
- Assessment of the impact of the intervention on productivity.

### 3.1. The Intervention Setting

The ISA sugar mill is a major operation located in Chichigalpa, Nicaragua, a known CKDnt hotspot [26,27,28]. The mill produces refined sugar, ethanol, rum, and power for the grid. ISA has a fully equipped hospital and laboratory that attends to workers and their family’s health needs (free of charge) and hence likely captures all hospitalized AKI in the workforce. There is also a fully staffed occupational health department. Around 3000 manual field laborers are employed in the cultivation and harvesting of sugar cane, the majority hired for the 6-month harvest period, but many are hired throughout the year for specific manual jobs as needed. Workers are not migrants and are drawn from many small communities in the area. The climate in northwestern Nicaragua during the harvest months of November–April is hot and relatively humid. Temperatures in the sugarcane fields rise rapidly and reach about 34 °C by about 10 a.m. (wet bulb globe temperature–WBGT, 30 °C) and 37 °C at 2 p.m. (WBGT 31 °C) [14].

In general, to be hired, an applicant must have a serum creatinine (SCr) <1.3 mg/dL (men) and <1.0 mg/dL (women) measured immediately pre-harvest. The mill’s operating prevention program focused primarily on the burned-cane cutters, who they believed to be the highest risk work group. The program included fixed-location base stations at each cutting area, where a reserve water supply and a shade tent were located. Health promoters moved around the work in the fields to identify workers with symptoms from heat exposure as well as, when requested, to take insulated personal water containers to the base station to refill them and carry them back to the worker. Mobile health clinics, staffed by a doctor, nurse, lab technician, and supervisor, rotated between active worksites attending most specifically the burned-cane-cutter groups.

The existing ISA prevention program was designed to address the physical demands of work required of the manual laborers. Key components were: (1) acclimatization for a 2-week period at the start of harvest; (2) stopping work at noon or as shortly after as possible; (3) regular rest breaks that varied by job type; and (4) a shade tent along with a water reservoir and electrolyte solution for each work group.

### 3.2. Designing the Intervention Study Assessment and Action Plan

In October 2017, the Adelante Initiative research team began a multi-year cohort study of workers at ISA to assess the standing prevention program. Field laborers were distinguished by job type and judgements of workload for each job type during harvest (i.e., a proxy for metabolic heat load). Otherwise, the laborers shared the same external work environment (climate), a common socioeconomic position (poverty), and living in similar places (rural coastal communities) with similar ambient exposures.

The overall study design included the following [14]:

- Characterization of the study population and their work environment demands, followed over multiple years, with
- Regular health assessments that included evaluation of kidney function pre- and post-harvest;
- Continuous assessment of the organization, implementation, and oversight of the intervention program and its communication to all levels; and
- A plan for recommending improvements to the program and their implementation.

**Figure 1:**
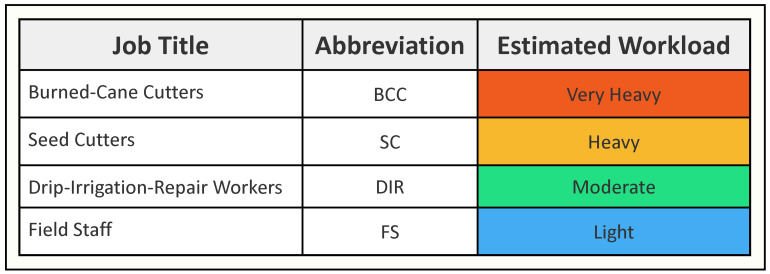
Job Categories and their Harvest 1 estimated workload.

Different job groupings were selected for the intervention assessment, targeting burned-cane cutters (BCC) who harvest cane, seed cutters (SC) who cut green cane into segments and bundle it for planting, drip-irrigation-repair workers (DIR) who continuously repair buried irrigation tubing, and field support staff (FSS). These groups were selected after discussions between ISA and the research team to represent, respectively, very heavy, heavy, moderate, and light physical workloads. This provided a means to examine the combined importance of external and internal sources of heat stress associated with the different jobs (Figure 1).

### 3.3. Timeline

The intervention implementation study began by assessing the existing prevention program over one harvest period (Harvest 1, H1). Baseline data were collected on a total of 525 participants at time of pre-harvest hire, with end-harvest data collected on the same individuals. In subsequent years, all participants who were rehired were included, supplemented by a number equal to those who did not return for a second or third year. Current work practices and working conditions were observed by the field team and implementation problems identified. Results were compiled and reported to ISA, recommendations were made for improvements for the following harvest (H2), and a plan was devised for monitoring implementation of all prevention aspects by the research industrial hygienist.

## 4. Intervention Program Assessment Results

### 4.1. Health Assessments

#### 4.1.1. Baseline-HI Health Assessment

**Figure 2:**
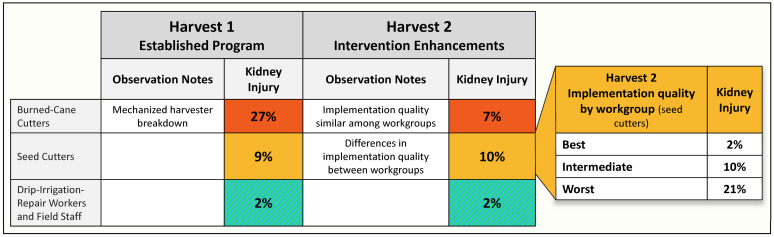
Change in cross-harvest kidney injury between Harvest 1 and Harvest 2. Significant workgroup differences only seen within seed-cutter job type (adapted from reference [15]).

Results from our H1 assessment determined that despite the ongoing prevention efforts, those estimated to have the very heavy and heavy workload had, respectively, a 13-fold (BCC) and a 4.5-fold (SC) risk of kidney injury compared to workers in the lower workload categories as shown in Figure 2 [14].

#### 4.1.2. H2 Health Assessment

The second-year results demonstrated a reduction of 72% incidence of kidney injury over the harvest among burned-cane cutters [15]. While the focus on burned-cane cutters greatly improved their situation, the H2 assessment demonstrated the SC workers appeared to show no improvement, while the other groups remained stable, with very little kidney injury (Figure 2) [15].

#### 4.1.3. H3 Health Assessment

**Figure 3:**
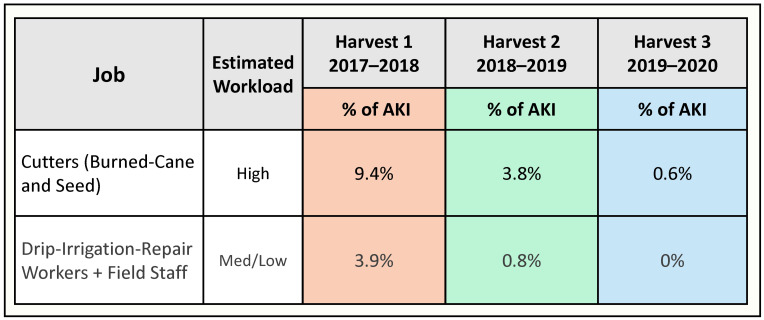
Incidence of hospitalized acute kidney injury (AKI) across three harvests. Percentages based on all sugarcane workers active in each job type.

Biological analysis results have been delayed due to the COVID pandemic. Fortunately, a retrospective evaluation of the incidence among active sugarcane workers of hospitalized AKI due to heat illness at the end of H3 was possible. Hospitalization related to heat illness is known to be associated with CKD, and it was thought the AKI events recorded at the ISA hospital could serve as an appropriate hard endpoint to guide ongoing intervention improvements. The findings (Figure 3) were encouraging, with drastic reduction in hospitalized AKI (94% in H3 as compared to H1).

### 4.2. Prevention Program Assessment

#### 4.2.1. H1 Baseline Program Assessment

Although shade tents and water resupply were largely in place as planned, these were observed to be inconvenient or inaccessible to the moving workers. Health promoters did help resupply workers with water but on request only. When on break, workers were often observed resting with no nearby shade available, nor did they necessarily rest for the intended periods. The existing program was best organized for the BCC group whose workday finished at noon, while fewer resources and rest periods were provided to the SC and DIR groups, both of which worked longer days. An unforeseen disruption occurred: two mechanical harvesters failed during the height of the harvest, leading the mill to suddenly hire ~160 new cutters to replace the machines. The mill discovered it did not have resilience built into its occupational health systems, and the resources (tents, coolers, support staff) were not expanded but either reallocated between groups or were altogether absent from some worksites during the hottest months of the year.

#### 4.2.2. H2 Intervention Enhancements

##### Recommended Improvements

While the elements of the Adelante Intervention could be considered conceptually simple in design and underlay the existing ISA program, the latter was missing key features for successful implementation. In brief, the recommendations for improvements included (a) assuring sufficient and appropriately convenient shade along with (b) easy access to adequate water/electrolyte solutions to maintain proper hydration, combined with (c) a revised plan and schedule for rest breaks to adequately recover from overly demanding work. The revised prevention program was to be implemented in all field settings, not just for BCC.

**Figure 4:**
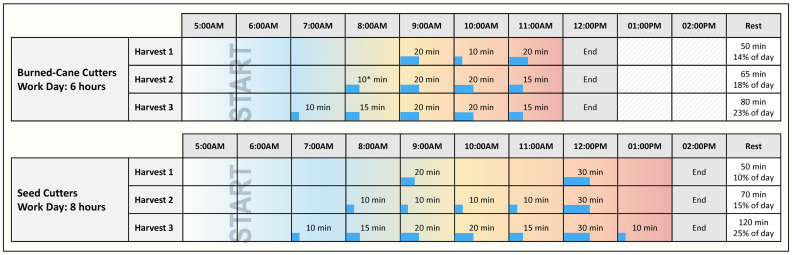
Rest schedule changes for burned and seed cane cutters: Existing (Harvest 1) and enhanced rest schedules observed (Harvest 2, Harvest 3), each providing more frequent and earlier rest periods. * Rest period at 08:00 a.m. in Harvest 2 was increased to 15 min for the last month of the harvest.

*Breaks overview*: Evidence-based best practices developed by the U.S. military [29] suggested that sufficient recovery time required enhanced rest/work schedules. Until systematic study of necessary rest frequency and duration could be performed, an initial proposal to increase number and duration of breaks was adopted to enhance rest for both BCC and SC (Figure 4).

**Figure 5:**
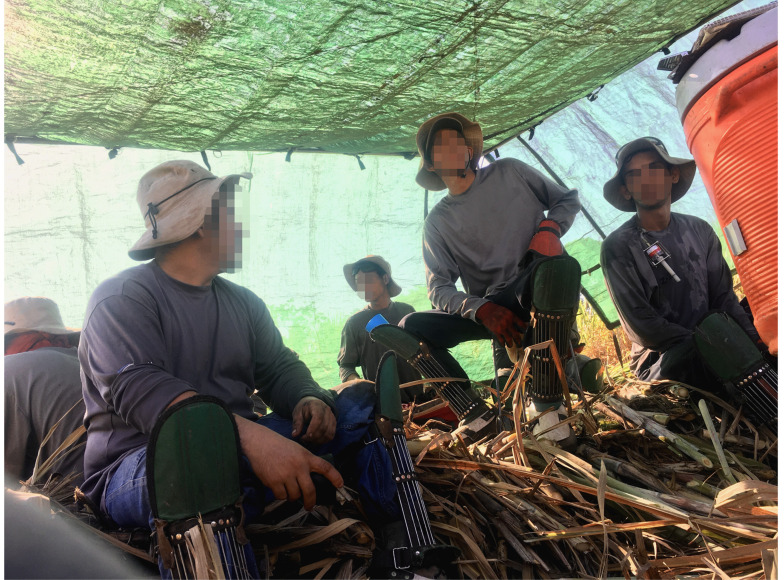
Lightweight shade tents provided sufficient space for 15 workers but were easily portable to permit moving along the workface.

*Shade overview*: In the absence of natural shade, additional shade tents were needed to avoid overcrowding in each. Furthermore, these needed to be light for easy movement throughout the day to keep close to the mobile workforce. Laborers needed to be provided with stools for comfortable rest and recovery (Figure 5).

*Hydration overview*: All field workers and staff needed easy access to sufficient potable water and electrolyte solutions in shaded coolers for regular refilling. Refill stations were to be set up throughout the work area in the shade tents and moved with the tents to keep close to the workforce (Figure 5). Adequate water and electrolyte solution reserves were to be carried on transport buses to meet the daily need should refill stations be depleted.

The program collaborators agreed that every effort be made to implement the intervention fully. All understood that if components were not well implemented, and results were poor, we could erroneously conclude that the intervention was ineffective because it was infeasible or designed poorly when it could simply have failed because it was not sufficiently adhered to. Therefore, equal emphasis was placed on intervention implementation along with intervention design, which resulted in a systematic plan to assess both the enhancements and the implementation of the intervention.

##### H2 Assessment of Improvements

The assessment indicated overall success of the enhanced intervention elements for the BCC group with almost a three-fourths reduction in kidney injury and stable, low levels for the DIR and staff groups (Figure 2). However, the lack of improvement for the SC group was a surprise. Fortunately, the research industrial hygienist’s observations provided a likely explanation. A review of his field notes identified no differences between burned-cane-cutting workgroups but a notable difference among the three seed-cutting workgroups. Supervisors and health promoters consistently paid more attention to complying with all the elements of the implementation in all BCC groups, while there was more mixed attention to details for the SC groups. Successful implementation was seen in only one of the SC subgroups, while in the two others, there was low consumption of water and electrolyte solution reported by workers, and shade tents were not well managed. One supervisor believed the seed cutters did not require the protections and apparently paid little attention to implementation (subgroup 3 “worst”), while another was quite the opposite, with diligent application of the intervention elements (subgroup 1 “best”). These differences may have been present in Harvest 1, but no records of subgroup differences were recorded.

#### 4.2.3. H3 Further Intervention Enhancements

##### Recommended Improvements

H2 formal assessment of workload began in H2 using Polar^®^ heartrate monitors to measure %HRmax and to estimate core body temperature. Observations were planned over two harvests. In H2, this workload assessment was piloted with 12 BCC male workers (average age 36), and 13 SC workers, including three females (average age 28), who were tested over a full workday in March. Greatly enhanced participant numbers were planned for H3 to include different months as well. Preliminary findings showed that core body temperature for both cutter groups commonly rose rapidly and was close to maximum by 7:00 a.m. It was decided to implement earlier rest in H3 and to expand total daily rest time for both BCC and SC, resulting in both types of cutters having approximately equal total resting time per workday (Figure 4).

Furthermore, in advance of Harvest 3, the research team together with the mill’s OSH and human resources offices planned a systematic assessment of implementation and what barriers to implementation remained. For this, a formal intervention implementation assessment tool was piloted by both groups in Harvest 3, with plans for parallel independent assessments by the ISA-OSH and the research team.

##### H2 Assessment of Improvements

**Figure 6:**
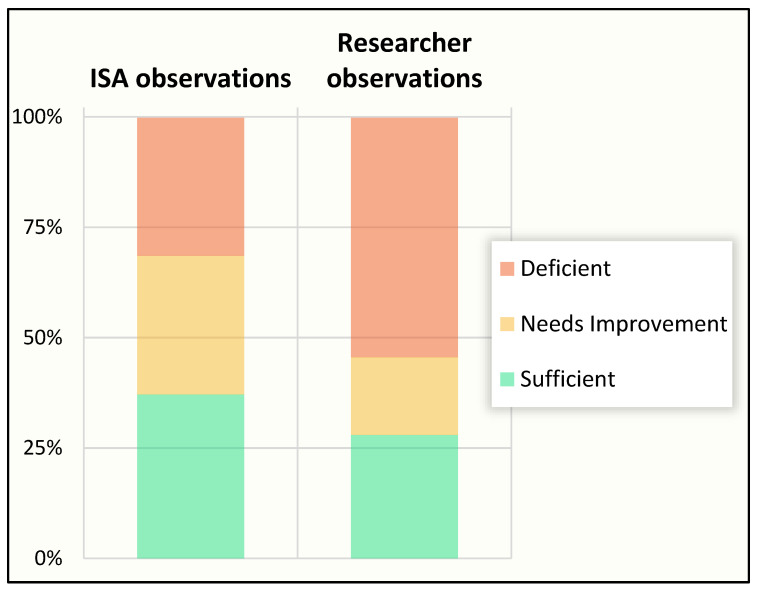
Summary of independent assessments of H3 intervention implementation.

Overall, results were similar from data collected by ISA OSH and the researchers in the period prior to the COVID-19-related restrictions when both teams used the same structured intervention implementation assessment tool (Appendix A provides an example used for the burned cane cutting job) during the limited time for observations (Figure 6). Across the jobs and settings, only about one-quarter of the prevention program demonstrated “sufficient” implementation of the full program. A notable outcome of this finding was that formal assessment by the mill’s OSH personnel led to their own documentation that their goals were not being attained.

The recorded details provided ISA staff with specific information on what aspects of the intervention were most difficult to deliver consistently. Parts of the intervention that could be improved were identified, specifically related to insufficient supplies of shade tents, thermoses, and latrines; use of cups for the electrolyte solution to better assess quantity consumed; and improved availability of the electrolyte solution and water reserves along with a scheme for distribution of hydration to places with natural shade.

Based on the H3 intervention evaluation, ISA acquired more supplies (awnings, hydration reserves, latrines) for each working group. Early in H4, ISA reported that the impact on the mill’s finances of the pandemic, plus two hurricanes striking early in the harvest, meant that they could not incorporate all the improvements planned.

In addition, after all parties were debriefed, it was evident that the quantitative implementation assessment tool that was being used was overly complicated and required too much effort to allow it to serve as a sustainable tool to track the implementation over the coming years. An improved and simplified tool was developed for H4, providing a user-friendly form to collect implementation details for the quality of the different elements of the water-shade-rest program.

## 5. Organizational Management Assessment

Concurrent with the planned implementation assessment during H3, an organizational management assessment was undertaken [30]. This assessment, focusing on middle management down to field supervisors, detected organizational barriers and proposed solutions. Among the findings was the observed tension between production and health. While field supervisors and support staff needed greater involvement to “own” the intervention, they experienced a sense of task overload in assuring adequate productivity (primarily due to the withdrawal of several field support staff after H2).

Despite the perception among all that top management was highly committed and the fact that middle management generally expressed a sense of pride in the intervention, the primacy of production goals appeared to be differently prioritized by organizational members along the management chain. The field supervisors and likely workers could have received contradictory messages about what was expected of them and in what ways they were “rewarded” by the workplace culture. This suggested that while worker safety was a key indicator for mill management, a culture that had prioritized productivity for many years meant some supervisors continued to perceive that the intervention metrics were not equivalently weighted to other performance indicators.

The piecework pay system was also identified as a structural factor likely undermining time devoted to heat-related safety efforts although the difficulty of implementing changes to this pay system was acknowledged. Piecework has been established as a chronic problem that confronts workers when trying to balance immediate income against longer-term ill effects [31,32,33,34].

The organizational assessment led to recommendations for enhanced education and messaging efforts at all levels and clear inclusion of health metrics for staff performance assessment so that the incentives and value of health and production can be adequately balanced. A participatory approach in the implementation that included worker inputs was recommended to foster not only compliance but meaningful involvement at all levels.

The response by the mill to these recommendations has been a commitment to address the need for adequate resources allocated to the field implementation and education of workers and supervisors and appropriate management incentives designed to ensure worker health is adequately prioritized not only by leadership but throughout the management chain. At present, the mill has ensured that 15% of the key performance indicators for the field supervisor performance assessments are on OSH metrics, emphasizing the importance of heat-related practices for employees, so it is clear to employees that this is more appropriately balanced with other considerations. The mill is also working to improve piece-rate-associated pressures by considering some form of day rate for each worker. Whether this would be a sufficient change to reduce harm is yet to be assessed.

## 6. Economic Assessment of Intervention

Alleviating heat-related kidney failure involves a substantial investment on the part of businesses that employ workers where jobs include risk of contracting illness due to working conditions. Enterprise leadership appears to readily assess the costs of health and safety controls without having a ready way to account for the costs of inaction. However, the returns on these investments, in addition to those derived from satisfying an employer’s moral obligations to care for the workforce, can be significant, as productivity can increase, turnover can decrease, and costs related to medical care can be reduced.

A return-on-investment (ROI) analysis was undertaken to examine evidence in this setting. The ROI estimation relied on an application of a generalized framework for ROI assessment of workplace healthcare interventions developed by the RAND Corporation [35]. To determine benefits and costs, we relied on data provided by ISA, interviews with ISA medical, human resources, and accounting staff members added to data gathered from secondary sources to calculate an estimated value of the returns [36]. Costs were estimated for medical care, intervention equipment and supplies, lost hours, and reduced productivity associated with CKDnt. Estimates of the value of sugar cane per ton were based on prevailing costs during the reference period (2017–2018) as calculated by macrotrends.net [37]. Benefits of the intervention were estimated based on savings from reduced turnover costs, increases in labor productivity, and reduced medical costs.

Findings from this examination of the returns on ISA’s rest, shade and water intervention have been promising, showing that for every USD 1.00 ISA invested in its water, rest, and shade regimen, it gained an additional USD 0.22 in return, or a return on investment (ROI) of 22%. A longitudinal study is planned to confirm how durable these gains are over a longer timeframe as temperatures and sugar cane prices fluctuate.

## 7. Lessons Learned

The limitations observed in the initial implementation demonstrate that the effectiveness of an intervention cannot be adequately assessed until implementation is also considered. This highlights the importance of explicitly including implementation evaluation in any intervention program. In this review of an evolving intervention, we set out to address most relevant aspects. We have described the learning curve for the various stakeholders involved, recognizing that this must continue as the intervention develops to achieve sustainability. Engaging all stakeholders improved the likelihood that necessary adaptations could be imagined, implemented, and systematically assessed. Recognizing that enterprises are in business to achieve economic success, we found it important to include economic assessment of an intervention at an early stage to show as soon as possible how such efforts affect the bottom line. In the process of this examination, we identified several lessons for successful and practical interventions both generally and with specific lessons for this setting.

### 7.1. Study Design

We began our engagement by considering possible options for designing this intervention and its formal assessment. Logistic challenges would come from many directions (civil unrest, natural disaster, difficult terrain, on-the-fly changes, and equipment failures), with a direct impact on worker protections. Added to these was a 125-year history of an industry operating in a low-regulatory environment and in the presence of an ineffective labor organization. These considerations impacted what was feasible for intervention design, assessment of that intervention, and interpretation of findings.

While the ideal of a clinical trial-like design and less so a randomized one was a starting point, we recognized how unfeasible this was in this setting. Ethically, no one could be denied the access to the prevention measures prescribed, especially as it is already well accepted that a rest, shade, and water intervention for heat stress at work is necessary. Practically, there was no logical way to explain to one subgroup why they were receiving less protection than their coworkers. Finally, this mill and workforce needed little to no convincing of the need for such programs in terms of health outcomes, so the practical value of demonstrating differences between treatment and control groups was nil. Logistically, we determined it was infeasible for the mill to provide different versions of the intervention or the same introduced at different intervals, such as in a stepped-wedge design. Putting multiple intervention elements into successful operation over a 70,000-hectare supply chain while adjusting the fundamentals for several at-risk job categories would be a complex task in any setting. Therefore, we settled on an observational before and after study as the appropriate approach.

### 7.2. Study Design Elements

#### 7.2.1. Dropouts

**Figure 7:**
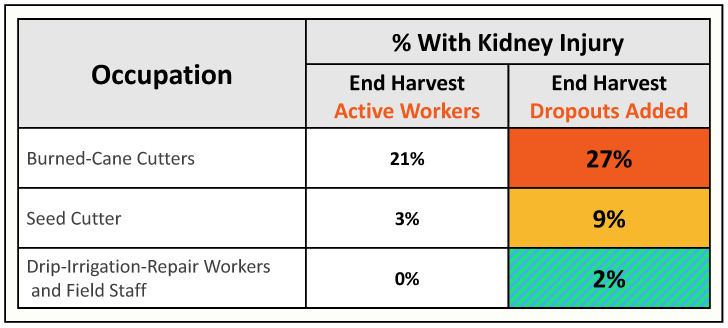
Baseline cross-harvest rates of kidney injury by job type with and without dropouts (adapted from reference [14]).

The study elements themselves required careful forethought and planning. Any longitudinal cohort analysis can be expected to lose participants over the course of time. This can add substantial penalty to interpretation of findings due to the healthy worker survival effect [38]. In this setting, a major effort was organized to collect contact information and to contact the 20% who did not attend the final annual assessment. We succeeded in reaching >90% of these “dropouts”, resulting in a more complete picture of the H1 results as shown in Figure 7. An effort continues to develop a system for identification of dropouts when they happen, for example, with a real-time system linking ISA OSH and HR to identify why a worker drops out.

#### 7.2.2. Appropriate Exposure Assessment

The ISA prevention program had been established based on observations and impressions that burned-cane cutters were performing the most physically demanding work. The other jobs covered a range of what was thought to be less demanding effort, but there was no objective evidence for work effort. Consequently, we introduced a work physiology method to study a sample of workers from a variety of manual job tasks. Quantitative workload assessments were based on heartrate measurements both to have objective measures of efforts and to provide physiologically relevant insights to how best to reduce the consequences of physically demanding work in hot environments. Preliminary results showed that both cutters of burned cane and cutters of seed cane were essentially the same. This led to improved attention to the intervention among the seed cutters. We are in the process of completing assessments of all major job tasks (a total of 10) over the course of 2–3 harvests. Early findings suggest that there are other jobs that may be as demanding as cutting but that had been more difficult to observe because these are less concentrated or carried out by small groups of workers over many planted acres. With a systematic approach to exposure assessment, we believe we will significantly improve the likelihood of finding the jobs most in need of attention and possible revision to work practices or work/rest cycles.

### 7.3. Implementation

From the outset the program, collaborators agreed that every effort be made to implement the intervention fully. All understood that if components were not well implemented, and results were poor, we could erroneously conclude that the intervention was ineffective because it was infeasible or designed poorly when it could simply have failed because adherence to the program elements were insufficient. Therefore, equal emphasis was placed on intervention *implementation* as well as intervention *design,* which resulted in a systematic plan to assess both the enhancements and the implementation of the intervention.

As the study proceeded, despite clear and specific endorsement of the intervention enhancements from mill senior leadership, we learned that successful implementation was impeded by insufficient engagement or understanding throughout the chain of command. The field staff were found to have different views about the importance of the intervention, with some fully committed and others not understanding the importance of their engagement. In response, the mill incorporated full participation in the intervention into job-performance assessments to provide practical motivation for the prevention program to be a priority. Development of an instrument for systematic implementation assessment was identified, and the instrument was introduced in H3 and continues to undergo revision to provide ongoing assessment. An early example of the success of this was the consistency in findings from the ISA and from the researcher team’s separate assessments (Figure 5). This has resulted in enhanced commitment to the value of organized and continuous assessment as is the case for measurements of productivity.

The H1 assessments of health and of the ISA prevention program practices led to the recognition that most of the necessary elements were present. However, there were major disconnects between the principles underlying the program and the observed practices. We were able to identify the principles for specific improvements called for in H2 and H3 to maximize likelihood that the goals of sufficient shaded rest and maintaining adequate hydration would be achieved. These consisted of:

- Mandated more frequent and earlier rest breaks;
- Breaks under adequate natural shade or tents that are designed to be moved easily to accompany the mobile workforce throughout each day. Tents are constructed from a netted fabric, open on two sides to provide adequate ventilation, and provided with stools for seated, shaded rest;
- Provision of potable water and electrolyte solution in tents kept close to the working area, therefore made easily available for ready access during prescribed rests and throughout the workday.

Adequate access to safe sanitation in the field, especially for women, who were thought to be limiting water intake to avoid sanitary breaks in the open field, was also prioritized.

### 7.4. Management Assessment

It has repeatedly been demonstrated that the workplace culture and the quality of employer–employee relations greatly affect perceptions between workers and supervisors and prevention behaviors as recently shown in industrial agricultural settings in Florida [32]. Work continues at ISA focused on identifying disconnects, perceptions, beliefs, and other barriers, such as the feeling of task overload that accompanied reorganization of field support for interventions. By formalizing the implementation assessment, challenges were depersonalized, making them plausible and addressable. This created positive engagement from the mill’s human resources and occupational health departments in committing to address messaging and operational gaps that have been contributing to inadequate implementation.

### 7.5. Managing on Exposures and Health Outcomes

It is crucial to nurture a culture of health and build an organizational safety climate [35] that is integrated into the more traditional management on productivity. Providing evidence on how the health of the workforce is better served when focus includes attention to adverse exposures that can be managed in the occupational setting is essential [35]. Such a focus is likely to have a greater success and subsequent impact on outcomes of concern than efforts at behavioral modification aimed at workers’ private lives that instead address risks, such as smoking or alcohol consumption.

### 7.6. Communication

Effective communication between the study team and all levels of an organization, from the labor force to the executive level, especially being transparent about all findings, is a priority. Documenting impacts through health and financial metrics is essential in order to encourage an employer to focus on controllable exposure risks that could impact production. Regular, continuous transparent communication with senior management promotes an open-mindedness in addressing any potential risk. People tend to become invested when they are in control of an issue that had formally only brought negative attention and consternation.

### 7.7. Onsite Researchers

The ongoing presence of researchers on site allows for consistent engagement and communication that can result in positive synergies, for example, between enterprise departments, especially when department leaders have not previously needed to collaborate. This was the case here for the OSH and human resources departments. Consistent site visits and communication with workers separate from the workplace setting is invaluable in permitting issues to be raised and addressed proactively. Employers who are committed to the effort, as has been the case here, understand this.

## 8. Conclusions

Within occupational health, the field of intervention and implementation research is in its infancy.

Designing, effectively implementing, and assessing both health impact and implementation quality is a resource intensive endeavor that requires a transdisciplinary approach. No one discipline can unpack the barriers and biases, propose how to address those from a management and implementation perspective, and then identify determinants of how improvements can be conscientiously and consistently carried out.

An inadequately implemented intervention can lead to one of or a combination of the following conclusions:

The intervention is poorly designed;

The intervention is poorly implemented;

The outcomes persist, and therefore, there must be additional relevant cause(s).

The last of these can be pernicious since inadequate implementation can lead to questioning allocation of finite resources and remove focus from an addressable risk. Without adequate attention to implementation support and assessment, there is always a risk of misunderstanding the lessons and losing a key opportunity to address a documented worker health issue.

## Data Availability

Only few data were created or analyzed in this study [hospitalized AKI and intervention implementation]. The new data are available on request from the corresponding author. Those data are not publicly available due to privacy restrictions. Otherwise, no new data were created or analyzed in this study and therefore data sharing is not applicable to these other data.
